# Supplementary material for: Tissue-scale tensional homeostasis in skin regulates structure and physiological function
Source: Commun Biol. 2020 Oct 30;3:637. doi: 10.1038/s42003-020-01365-7 (PMC7603398; doi:10.1038/s42003-020-01365-7)
Supplement: Supplementary file 2 — Reporting Summary [file 42003_2020_1365_MOESM2_ESM.pdf]

nature research

Corresponding author(s): Shun Kimura, Ayako Tsuchiya, Miho Ogawa, Miki Ono, Nao Suda, Kaori Sekimoto, Makoto Takeo & Takashi Tsuji

Last updated by author(s): Sep 15, 2020

Reporting Summary

Nature Research wishes to improve the reproducibility of the work that we publish. This form provides structure for consistency and transparency in reporting. For further information on Nature Research policies, seeAuthors & Referees and theEditorial Policy Checklist .

Statistics

For all statistical analyses, confirm that the following items are present in the figure legend, table legend, main text, or Methods section.

n/a	Confirmed	
☐	☒	The exact sample size (*n*) for each experimental group/condition, given as a discrete number and unit of measurement
☐	☒	A statement on whether measurements were taken from distinct samples or whether the same sample was measured repeatedly
☐	☒	The statistical test(s) used AND whether they are one- or two-sided<br>*Only common tests should be described solely by name; describe more complex techniques in the Methods section.*
☐	☒	A description of all covariates tested
☐	☒	A description of any assumptions or corrections, such as tests of normality and adjustment for multiple comparisons
☐	☒	A full description of the statistical parameters including central tendency (e.g. means) or other basic estimates (e.g. regression coefficient) AND variation (e.g. standard deviation) or associated estimates of uncertainty (e.g. confidence intervals)
☐	☒	For null hypothesis testing, the test statistic (e.g. *F*, *t*, *r*) with confidence intervals, effect sizes, degrees of freedom and *P* value noted<br>*Give P values as exact values whenever suitable.*
☐	☒	For Bayesian analysis, information on the choice of priors and Markov chain Monte Carlo settings
☐	☒	For hierarchical and complex designs, identification of the appropriate level for tests and full reporting of outcomes
☐	☒	Estimates of effect sizes (e.g. Cohen's *d*, Pearson's *r*), indicating how they were calculated

Our web collection on statistics for biologists contains articles on many of the points above.

Software and code

Policy information about availability of computer code

Data collection	ZEN blue eddition, ZEN black eddition, QuantStudio 12K Flex
Data analysis	ZEN blue eddition, ZEN black eddition, QuantStudio 12K Flex, Microsoft Excel, Imaris 8, Image J, and BellCurve for Excel

For manuscripts utilizing custom algorithms or software that are central to the research but not yet described in published literature, software must be made available to editors/reviewers. We strongly encourage code deposition in a community repository (e.g. GitHub). See the Nature Research guidelines for submitting code & software for further information.

Data

Policy information about availability of data

All manuscripts must include a data availability statement. This statement should provide the following information, where applicable:

- Accession codes, unique identifiers, or web links for publicly available datasets
- A list of figures that have associated raw data
- A description of any restrictions on data availability

All data are available from the corresponding authors upon reasonable request.

Field-specific reporting

Please select the one below that is the best fit for your research. If you are not sure, read the appropriate sections before making your selection.

☒ Life sciences ☐ Behavioural & social sciences ☐ Ecological, evolutionary & environmental sciences

For a reference copy of the document with all sections, see nature.com/documents/nr-reporting-summary-flat.pdf

Life sciences study design

All studies must disclose on these points even when the disclosure is negative.

Sample size	We did not perform the sample size calculation.
Data exclusions	We did not exclude any data from the analysis.
Replication	We repeated same experiments at least 3 times and confirm reproducibility.
Randomization	We allocate samples equally and randomly into individual experimental group.
Blinding	We were blinded to sample allocation and during analysis.

Reporting for specific materials, systems and methods

We require information from authors about some types of materials, experimental systems and methods used in many studies. Here, indicate whether each material, system or method listed is relevant to your study. If you are not sure if a list item applies to your research, read the appropriate section before selecting a response.

Materials & experimental systems

n/a	Involved in the study	
☐	☒	Antibodies
☒	☐	Eukaryotic cell lines
☒	☐	Palaeontology
☐	☒	Animals and other organisms
☒	☐	Human research participants
☒	☐	Clinical data

Methods

n/a	Involved in the study	
☒	☐	ChIP-seq
☒	☐	Flow cytometry
☒	☐	MRI-based neuroimaging

Antibodies

Antibodies used

Anti-alpha smooth muscle Actin antibody, Abcam, Ab5694
Anti-c-Jun antibody [E254], Abcam, Ab32137
Anti-Cytokeratin 10 antibody, Abcam, Ab9026
Anti-Cytokeratin 5 antibody, Abcam, Ab52635
Claudin 1 antibody, Thermo Fisher Scientific, 71-7800
Anti-Collagen I antibody, Abcam, ab34710
Anti-Collagen IV antibody, Abcam, ab6586
Anti-Filaggrin antibody [SPM181], Abcam, ab17808
Anti-Integrin alpha 2 antibody [EPR5788], Abcam, ab133557
Anti-Integrin beta 1 antibody, Abcam, ab30388
Anti-Ki67 antibody, Abcam, ab156956
Anti-Collagen III antibody, Abcam, ab6310
Anti-Collagen VII antibody, Abcam, ab6312
Anti-Collagen XVII antibody, Abcam, ab184996
Phalloidin-Alexa594 conjugate, Thermo Fisher Scientific, R415
Alexa Fluor™ 488 Phalloidin, Thermo Fisher Scientific, A12379
Anti-Mkl1/MRTFA antibody, Abcam, ab113264
Wheat Germ Agglutinin, Alexa Fluor™ 488 Conjugate, Thermo Fisher Scientific, W11261
Alexa Fluor 594 donkey anti-rabbit IgG, Thermo Fisher Scientific, A21207
Alexa Fluor® 594 Donkey Anti-Mouse IgG (H+L) Antibody, Thermo Fisher Scientific, A21203
Alexa Fluor® 488 Donkey Anti-Rabbit IgG (H+L), Thermo Fisher Scientific, A21206
Alexa Fluor® 488 donkey anti-mouse IgG (H+L), Thermo Fisher Scientific, A21202
Alexa Fluor 633 goat anti-rat IgG(H+L), Thermo Fisher Scientific, A21094

Validation

We chose primary antibodies according to the manufacture's validation.

Animals and other organisms

Policy information about studies involving animals; ARRIVE guidelines recommended for reporting animal research

Laboratory animals	Experiment used C57BL/6N (Japan SLC Inc.). Seven to nine-weeks old female mice were used.
Wild animals	None
Field-collected samples	None
Ethics oversight	All the experimental procedures using animals were approved by the Institutional Animal Care and Use Committee of RIKEN Kobe Branch

Note that full information on the approval of the study protocol must also be provided in the manuscript.
